# Tea Tree Oil Microemulsion-Gel-Strengthened Soy Protein Isolate Composite Films: A Multifunctional Active Packaging System

**DOI:** 10.3390/gels12060460

**Published:** 2026-05-25

**Authors:** Minghang Zhao, Yulu Xie, Pengbo Wang, Xuyu Hao, Yutong Xu, Dongyang Zhao, Zhengxiong Wang, Hao Chen

**Affiliations:** 1 SDU-ANU Joint Science College, Shandong University (Weihai), Weihai 264209, China; 202300700230@mail.sdu.edu.cn; 2 College of Marine Sciences, Shandong University (Weihai), Weihai 264209, China; 202300810180@mail.sdu.edu.cn (Y.X.); 202300630179@sdu.edu.cn (P.W.); xuyuhao@mail.sdu.edu.cn (X.H.); 202500810215@mail.sdu.edu.cn (Y.X.);

**Keywords:** soy protein isolate, gel film, tea tree oil microemulsion, active packaging, sustained release, mechanical properties

## Abstract

The development of stable and efficient essential oil delivery systems remains a persistent challenge in active food packaging applications. This research aimed to develop a multi-functional soy protein isolate (SPI)-based composite gel film integrating a tea tree oil micro emulsion (TME) via a microemulsion-in-gel approach, featuring sustained antioxidant release. The TME was first optimized using pseudo-ternary phase diagrams and exhibited excellent physicochemical stability. It maintained a droplet size ranging from 10 to 13 nm, with a polydispersity index (PDI) less than 0.2 under diverse stress situations (such as dilution, heat treatment, pH change, centrifugation, and 30-day storage). Afterward, TME-SPI composite gel films containing 1 to 3% TME were fabricated through solution casting and subsequent gelation of the protein matrix. The incorporation of TME markedly improved the properties of the gel film network. It raised the opacity by around 2.5 times, boosted the elongation at break to 144% (which is three times that of the control), and distinctively enhanced both water solubility and the water vapor barrier. Importantly, the 2% TME-SPI gel film exhibited sustained antioxidant activity from within the gel matrix, retaining more than 50% of its original 1,1-diphenyl-2-picrylhydrazyl (DPPH) scavenging activity after 72 h, significantly outperforming films containing free TTO. The microemulsion-in-gel approach was shown to be effective in creating SPI-based gel films that possess combined light-barrier characteristics, adjustable moisture resistance, improved flexibility, and extended antioxidant release. This offers a promising framework for the next generation of active food packaging. Furthermore, the composite gel films exhibited concentration-dependent antibacterial activity against Staphylococcus aureus, with the 3% TME-SPI film achieving an 82% inhibition rate, thus experimentally validating its active packaging potential.

## 1. Introduction

Global food packaging depends significantly on plastics derived from petroleum. This reliance has led to serious environmental concerns, such as microplastic contamination, which calls for urgent solutions [1]. Therefore, it is imperative to develop renewable and biodegradable substitutes.

SPI gel films fabricated from natural biopolymers represent a promising alternative. They are readily accessible and eco-friendly [2]. However, current SPI gel films frequently suffer from structural deficiencies. Typical issues include inadequate mechanical strength and suboptimal water vapor barrier properties. These limitations restrict their practical application in food packaging.

To enhance their functionality, active packaging systems have been created. These systems integrate bioactive elements having antioxidant or antimicrobial capabilities. Such gel films not only serve as a protective barrier but also actively regulate the food microenvironment, thereby effectively extending product shelf life [3].

At present, most active additives are hydrophilic substances, such as plant extracts, organic acids, and enzymes. Research on these additives is extensive due to their favorable compatibility with hydrophilic film matrices. Nevertheless, numerous hydrophobic substances also display outstanding biological activities. They possess extensive application possibilities [4]. Plant essential oils serve as a crucial instance. They are regarded for their natural source and wide-spectrum antibacterial characteristics.

Tea tree oil (*Melaleuca alternifolia* oil) is a representative hydrophobic essential oil [5]. It has a wide range of antimicrobial, anti-inflammatory, and antioxidant properties [6]. Integrating tea tree oil (TTO) into gel films is anticipated to impart antimicrobial capabilities [7]. However, owing to its hydrophobic nature, difficulties arise when TTO is directly incorporated into a hydrophilic gel matrix, such as those based on proteins or polysaccharides. Direct incorporation frequently results in problems. These involve non-uniform film structure, degraded mechanical characteristics, and burst release or loss of active ingredients during storage. These problems seriously undermine the performance of films. The challenge of integrating hydrophobic active substances into SPI gel films has restricted their practical use for a long time. Achieving the successful incorporation of hydrophobic compounds into hydrophilic matrices thus remains a central challenge.

To tackle this issue, researchers have investigated pre-treatment methods. These involve emulsification, micro-encapsulation, and nano-encapsulation [8]. These approaches aim to encapsulate hydrophobic active compounds prior to their incorporation into the film system. Among these, microemulsions are regarded as an optimal choice owing to their distinct structural advantages. Microemulsions are thermodynamically stable and possess extremely small droplet sizes, generally ranging from 10 to 100 nm. These features enable efficient encapsulation of hydrophobic tea tree oil in a nanoscale lipid phase [9]. This enables its even dispersion within the continuous SPI hydrogel network. Consequently, it aids in preventing the separation of phases and the burst release of active ingredients. It efficiently resolves the compatibility issue. Moreover, when the film is drying, the nanoscale lipid phase might form a meandering pathway structure. This framework can extend the diffusion route for water vapor molecules. It might thus improve the film’s water vapor barrier properties [10]. This partly makes up for the intrinsically low water-resistance of SPI gel films. This two-fold effect endows microemulsion technology with distinct value in the development of functional SPI gel films.

Based on this, integrating tea tree oil-loaded microemulsions into a protein gel matrix to construct a microemulsion–gel composite film presents a promising approach. This research validates the microemulsion-in-gel approach, which is capable of simultaneously utilizing hydrophobic active substances and improving the barrier properties of the film. However, comprehensive investigations regarding tea tree oil microemulsion–protein composite systems are still scarce. Specifically, a comprehensive physicochemical and functional description of them is absent. Critically, while the antibacterial properties of TTO are well-documented, demonstrating that this activity is retained and effective after incorporation into a complex SPI gel film matrix requires direct experimental validation. This is an essential film characteristic that cannot be inferred from the properties of individual components alone, as interactions within the matrix may diminish or, conversely, potentiate the activity.

In this research, a microemulsion of tea tree oil was formulated and optimized. Pseudo-ternary phase plots were employed. The stability of it under different stress circumstances was evaluated. A gel film having improved functional attributes was subsequently developed. This gel film employed the micro-emulsion and SPI as the film-forming matrix. The features of the film were described. These involved optical features, hydration properties, mechanical capabilities, and continuous antioxidant release. Moreover, the release kinetics of TME-SPI films were contrasted with those of films having free tea tree oil. This assessed the encapsulation effectiveness of the microemulsion system. This research validates the viability of the microemulsion-in-gel approach. It concurrently enhances barrier characteristics, boosts mechanical pliability, and attains continuous active release. This offers a novel approach for the development of high-performance multi-functional active packaging materials.

## 2. Results and Discussion

### 2.1. Characterization of Tea Tree Oil (TTO) Microemulsion (ME)

#### 2.1.1. Pseudo-Ternary Phase Diagrams and Dilution Stability

Pseudo-ternary phase diagrams are a widely recognized tool in the study of microemulsion formulations [11]. They represent the phase behavior of the oil phase, surfactant/cosurfactant mixture (Smix), and aqueous phase in a two-dimensional diagram. This visually pinpoints the area where a thermodynamically stable, single-phase microemulsion may be present.

**Figure 1:**
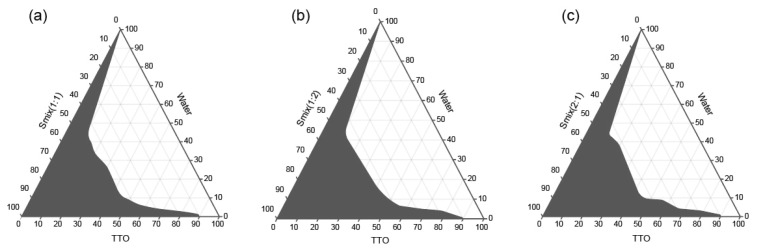
Pseudo-ternary phase diagrams of the Tween 80/ethanol/tea tree oil/water system at Smix ratios of (**a**) 1:1, (**b**) 1:2, and (**c**) 2:1. The shaded areas represent the single-phase microemulsion regions.

The pseudo-ternary phase diagrams indicated that all examined Smix ratios (1:1, 1:2, and 2:1) were capable of forming stable microemulsions with TTO. As shown in Figure 1a, every ratio generated a distinct microemulsion area, suggesting favorable microemulsion formation over a comparatively broad range of compositions. Quantitative analysis revealed that the areas of these regions were comparable, and all satisfied the fundamental requirements for microemulsion formation.

Based on these findings, the Smix with a Tween 80-to-ethanol ratio of 1:1 was selected for preparing the composite films. The microemulsion was required to act as a nanofiller in an SPI matrix under challenging circumstances, such as alkaline pH (9), elevated temperature (80 °C), and the requirement for beneficial interfacial interactions with SPI molecular chains. The 1:1 formulation provided a sufficiently large microemulsion region in the phase diagram, ensuring the preparation of a thermodynamically stable system. More importantly, its well-balanced surfactant composition was expected to promote a compatible and stable interface with the SPI gel matrix. This would enable the even dispersion of the nanodroplets in the protein gel network, safeguarding their structural integrity.

Thus, the pseudo-ternary phase diagram analysis provided a basis for formulation design. It verified that all ratios were capable of forming stable microemulsions. However, the final choice was refined by taking into account the practical requirements of film preparation. In contrast to traditional emulsification techniques, the micro-emulsion method presents distinct benefits in attaining homogeneous dispersion and enduring alkaline and thermal circumstances. Moreover, this approach can be extended to other hydrophobic bioactive substances and protein matrices.

**Figure 2:**
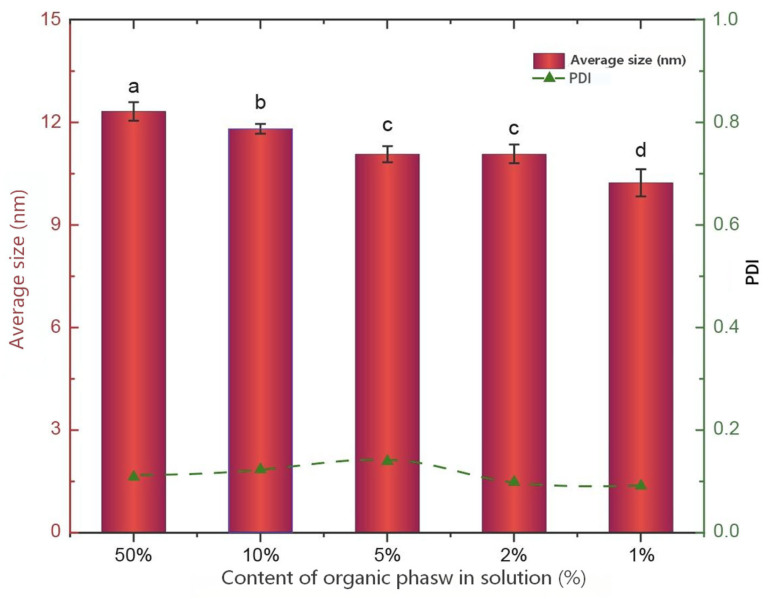
Average size and PDI of microemulsion at different dilution ratios. Different lowercase letters at the same time point indicate statistically significant differences (*p* < 0.05) between formulations.

The dilution stability of the prepared TTO microemulsion was evaluated across a range of dilution factors. As shown in Figure 2, when the microemulsion was diluted by factors of 2, 10, 20, 50, and 100, the mean particle size remained within a narrow range of 10–13 nm. The PDI remained consistently below 0.2 across all tested dilution ratios. Under the determined optimal dilution conditions, the mean particle size and PDI of the microemulsion were maintained at approximately 11.5 nm and 0.15, respectively. The nanodroplet structure remained stable across the entire dilution range examined, without obvious aggregation or phase splitting. Based on these outcomes, the formulation diluted by 50 times was chosen for systematic stability testing under thermal, pH, centrifugal, and storage circumstances. The microemulsion diluted by a factor of 10 was employed for antioxidant activity experiments.

The excellent dilution stability observed in this study can be attributed to the formation of a robust interfacial film around the oil droplets. This interfacial layer, made up of Tween 80 and ethanol, efficiently stops droplet aggregation, flocculation, and Ostwald ripening when diluted [12,13]. Compared with conventional emulsions and other essential oil microemulsion systems reported in the literature, the present formulation exhibited superior stability. Certain essential oil nano-emulsions have exhibited notable increases in particle size and PDI values greater than 0.3 following 50-fold dilution. In contrast, the current microemulsion kept the particle size under 13 nm and the PDI under 0.2 for all dilution ratios. This stability is further supported by analysis of the pseudo-ternary phase diagrams, in which the microemulsion regions extended toward the water apex, theoretically indicating the potential for infinite dilution without phase separation [14,15]. This combination of a robust interfacial film and favorable phase behavior ensures that the nanodroplets retain their size and uniform distribution upon subsequent incorporation into protein film matrices, thereby providing a solid foundation for fabricating composite films with enhanced functional properties.

#### 2.1.2. Stability Under Stress Conditions

**Figure 3:**
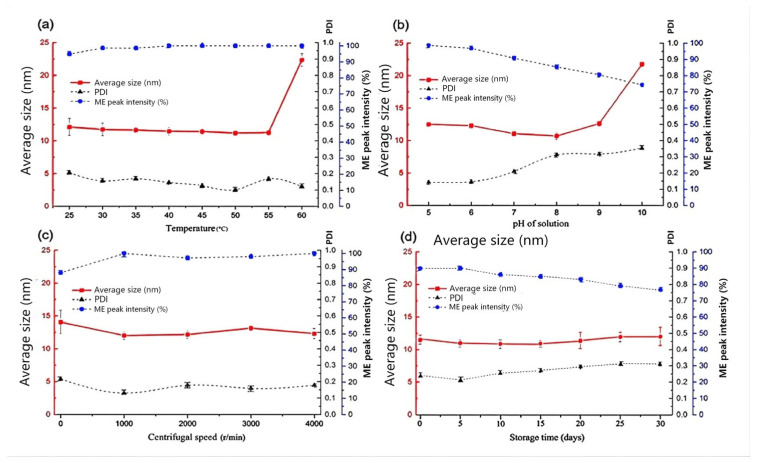
The stability of the TTO micro-emulsion diluted 50 times under various environmental stress conditions. (**a**) Thermal stability: Alterations in Average size (Z-average) and polydispersity index (PDI) when heating from 25 to 60 °C. (**b**) pH stability: alterations in particle size and PDI across the pH scope of 5–9. (**c**) Centrifugal steadiness: Influence of rotational velocity (1000–4000 rpm) on colloidal factors. (**d**) Storage steadiness: Alterations in particle dimension and polydisperse index (PDI) within 30 days at 25 °C.

The stability of the TTO-ME was systematically evaluated under various environmental stresses, including thermal, pH, centrifugal, and storage conditions (Figure 3). Throughout all the tests, the micro-emulsion kept outstanding colloidal integrity, where the particle size and polydispersity index (PDI) stayed within narrow intervals. These findings show that TTO-ME has strong physicochemical stability, offering a reliable foundation for subsequent film fabrication and functional performance.

As presented in Figure 3a, when the thermal treatment was carried out from 25 °C to 55 °C, there was minimal fluctuation in the particle size (12–14 nm) and PDI (0.1–0.2), and no significant changes were observed. Even at 55 °C, which is close to the film-forming temperature, the system remained stable, demonstrating favorable thermal resistance. The stability of the microemulsion with respect to pH was assessed within a range from 5 to 10 (Figure 3b). At a pH value of 5.16, the size of the droplets was 12.52 nm, and a slight opalescence was noticed. As the pH increased to 7.69, the mean size rose to 26.63 nm; nevertheless, the main population remained around 13.23 nm. At pH values of 8.00 and 9.05, the system reached a stable state, having average sizes of 10.71 nm and 13.63 nm, respectively, with the main intensity peak (Z-average of the principal population) at 10.10 nm and 11.53 nm. At a pH value of 10.01, the mean size grew to 21.75 nm, and the primary peak was at 13.57 nm. Notably, when the particle size exceeded 100 nm, partial demulsification was observed, and values above 1000 nm suggested the presence of impurities. In general, the micro-emulsion stayed relatively stable in the pH range of 7 to 9, showing minimal changes in size. At pH 9, which is the ideal state for the following SPI film creation, no phase segregation or notable size growth took place, validating its compatibility with the alkaline film-forming environment. As presented in Figure 3c, when the centrifugal speed rose from 1000 revolutions per minute to 4000 rpm, the size of the droplets stayed stable within the range of 10 nm to 15 nm, and the PDI remained under 0.3, without any visible phase separation, verifying the excellent mechanical stability and coalescence resistance of the microemulsion [15]. Figure 3d depicts storage stability spanning 30 days at 25 °C: the size of particles stayed stable (12–14 nm) for a minimum of 20 days. The PDI rose only marginally and stayed below 0.35, surpassing comparable antibacterial microemulsion systems [16,17].

In contrast to traditional emulsions or nanoemulsions reliant on ultrasonication, our microemulsion system presents unique benefits. Traditional emulsions usually need high-energy input, and they display droplet sizes larger than 100 nm, having wider size distributions and less stability when stored or when the pH varies. Ultrasonication-derived nanoemulsions, although attaining smaller droplets, frequently experience heat-caused degradation of sensitive bioactives and are short of thermodynamic stability. In comparison, our TTO-ME forms on its own, keeps ultra-tiny droplet sizes (10–15 nm) with a narrow PDI (<0.2), and endures thermal, centrifugal, and pH stresses without any structural damage. This robustness guarantees steady nanodroplet distribution within the protein framework, allowing for even film characteristics and continuous antioxidant liberation—benefits not easily attained through traditional emulsification methods.

To further contextualize the robustness of the TTO-ME system, it is instructive to compare its stability profile with those of recently developed essential oil microemulsion formulations. Yang et al. [15] reported that forsythia essential oil microemulsions exhibited particle sizes ranging from 20 to 80 nm depending on the surfactant ratio, with PDI values between 0.2 and 0.4; storage stability testing revealed noticeable size increases after only 15 days at ambient temperature. In comparison, the present TTO-ME system maintained particle sizes of 12–14 nm with a PDI below 0.35 over the entire 30-day storage period, with no evidence of phase separation, creaming, or significant size evolution. Furthermore, unlike the forsythia essential oil system, which exhibited marked pH sensitivity and underwent rapid coalescence below pH 6, the TTO-ME remained colloidally stable over a broad pH range (5–10), albeit with optimal performance observed between pH 7 and 9. This comprehensive stress resistance—encompassing thermal, centrifugal, pH, and long-term storage stability—positions the TTO-ME as a particularly robust carrier for incorporation into protein-based film matrices, where exposure to alkaline conditions (pH 9) and elevated temperatures (up to 80 °C) constitutes an integral part of the film-forming process. These results collectively confirm the viability of the microemulsion-in-gel approach and provide a solid foundation for its application in multifunctional active packaging materials.

### 2.2. Structure and Physical Properties of Composite Gel Films

#### 2.2.1. Morphology, Color, and Optical Properties

Variations in color and optical properties not only affect the film’s appearance but also impart functional light-barrier properties. As shown in Table 1, the incorporation of TME led to a concentration-dependent increase in both film opacity and total color difference (ΔE), whereas light transmittance decreased correspondingly. The neat SPI film exhibited an opacity of approximately 0.4. Although the inclusion of free TTO (1% T-SPI) did not notably change this value, the opacity rose gradually with a higher TME content, attaining 3.0 for the 3% TME-SPI film, which was approximately a 2.5-fold increase. Meanwhile, compared with recent studies, the color change observed here was more pronounced. Findings on comparable nanocomposite films indicated a ΔE rise of around 8–12 when a nanoemulsion was added, while the ΔE for the 3% TME-SPI film in this setup reached 20.28. This pronounced color change, characterized by an increase in yellowness (b*) and a decrease in lightness (L), is primarily attributed to the efficient Mie scattering of incident light by the uniformly dispersed nano-sized droplets (<15 nm) within the TME. Moreover, the non-ionic surfactant Tween 80 can position itself among SPI protein chains, affecting hydrophobic interactions and hydrogen-bonding networks. As a result, it further alters the film’s microstructure and light-scattering characteristics [18].

The pronounced changes in color and optical properties induced by TME incorporation can be understood by considering the dual role of the microemulsion components within the protein gel matrix. On the one hand, the nano-sized oil droplets (10–15 nm) serve as efficient light-scattering centers via Mie scattering, as their dimensions are comparable to or smaller than the wavelengths of visible light (380–780 nm). When uniformly dispersed throughout the film matrix, these nanodroplets generate a dense network of scattering interfaces that effectively deflect incident photons, thereby simultaneously increasing opacity and reducing light transmittance. On the other hand, the non-ionic surfactant Tween 80, which constitutes a substantial fraction of the TME formulation, is known to intercalate between protein chains within the SPI matrix. The hydrophilic polyoxyethylene (PEO) head groups of Tween 80 can compete for hydrogen-bonding sites along the protein backbone, partially disrupting the native protein–protein interactions and altering the supramolecular organization of the gel network. This surfactant-mediated modulation of the protein matrix microstructure further contributes to the observed changes in light-scattering behavior, as variations in the density and spatial distribution of scattering heterogeneities within the film directly influence its optical properties. The combined effect of nanodroplet scattering and surfactant-induced structural reorganization accounts for the substantially higher ΔE values (up to 20.28 at 3% TME loading) observed in this study relative to the ΔE range of 8–12 typically reported for nanoemulsion-containing composite films [18,19,20]. The resulting high opacity and low light transmittance endow the TME-SPI films with excellent light-barrier properties, enabling them to effectively block both ultraviolet and visible light and thereby provide passive photoprotection for light-sensitive foods (such as nuts, oils, and pigmented products), which broadens their potential application in active packaging [19,20].

#### 2.2.2. Optical Properties, Film Thickness, Hydration Behavior, and Mechanical Properties

**Figure 4:**
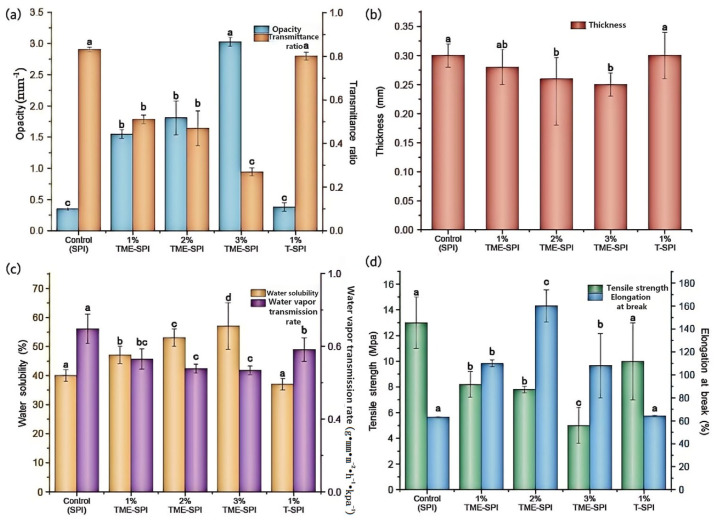
Physical and mechanical characteristics of SPI films having TTO microemulsion (TME) or unencapsulated TTO (T-SPI). Different lowercase letters at the same time point indicate statistically significant differences (*p* < 0.05) between formulations. (**a**) Opacity (mm^−1^ left axis) compared with Transmittance ratio (right axis); the inserted table sums up color parameters (L, *a*, *b*, ΔE). (**b**) film thickness (mm). (**c**) Solubility in water (WS, left-hand axis) (%) and Water vapor (WVP, right-hand axis). (**d**) Tensile strength (TS) (Mpa) and Elongation at break (EAB) (%).

The incorporation of the TTO microemulsion (TME) systematically altered the structure, hydration behavior, and mechanical properties of the SPI films (Figure 4) [21]. The alterations in diverse properties as the TME content rises show distinct trends and synergistic impacts.

##### Optical Characteristics: Opacity and Light Transmission

As depicted in Figure 4a, the addition of TME notably enhanced film opacity and simultaneously decreased light transmittance. The opacity values of both the control and the film containing free TTO remained approximately 0.4. In contrast, the opacity increased gradually with increasing TME content, rising from 0.4 for the control film to 3.0 for the 3% TME-SPI film, representing an approximately 2.5-fold increase.

The observed increase in opacity is primarily ascribed to the efficient scattering of incident light by the uniformly dispersed nano-sized droplets (<15 nm) within the TME [22]. When the size of the droplets is less than the wavelength of visible light and they are distributed uniformly, a large number of scattering interfaces are formed within the film matrix, which effectively hinders the transmission of light. In contrast to recent research, the enhancement in light-barrier characteristics attained in this study is more remarkable. For example, a recent study improved opacity by adding large particulate fillers, which frequently impair film flexibility [23]. In comparison, the present system utilizes nanodroplet scattering to enhance light-blocking ability while preserving the material’s flexibility. This distinction carries mechanistic significance. While large particulate fillers (typically in the micrometer size range) achieve opacity enhancement primarily through broadband scattering and absorption, they inevitably introduce stress concentration points that embrittle the film. In contrast, the nanoscale TME droplets (<15 nm) generate effective light scattering via the Mie mechanism without creating mechanically weak interfaces. The small droplet size relative to the wavelength of visible light (380–780 nm) ensures efficient scattering in the shorter-wavelength region while minimizing optical defects such as haze and macroscopic inhomogeneity. Moreover, the liquid nature of the oil cores—as opposed to the rigidity of conventional solid fillers—allows the nanodroplets to deform under mechanical stress rather than act as fracture initiation sites. This unique characteristic preserves the film’s flexibility and contributes, synergistically with the plasticizing effect of Tween 80, to the substantial enhancement of elongation at break. This improvement in optical properties demonstrates that the films possess enhanced physical light-blocking ability, making them appropriate for packaging light-sensitive food items like nuts, oils, and pigmented products [24]. 

##### Film Thickness

As shown in Figure 4b, the thickness of the neat SPI film was around 0.30 mm. The addition of 1% unbound TTO or 1% TME did not lead to substantial changes in this value. However, a clear decreasing trend in thickness was observed as the TME content increased. Specifically, the thickness decreased to 0.26 mm for the 2% TME-SPI film and further to 0.25 mm for the 3% TME-SPI film.

The decrease in film thickness at higher TME loadings can be attributed to the corresponding reduction in the SPI-to-total-solids ratio in the casting solution. This outcome is consistent with the typical behavior of composite systems, where the inclusion of a non-film-forming dispersed phase at the cost of the continuous matrix may result in a thinner dry film layer. The acquired thickness scope of 0.25–0.30 mm for every film is in agreement with the standard thickness stated for most biodegradable packaging films, validating the viability of the film-forming procedure [25]. This regulated and foreseeable change in thickness, without significantly modifying the macroscopic film sizes, might lead to slight changes in other physical characteristics such as mechanical strength and barrier functionality, which are addressed in the following sections.

##### Hydration Phenomenon: Hydrophilic Characteristics and Moisture-Blocking Attributes

Figure 4c reveals a distinct contrast in the hydration behavior of the films after TME addition. As the water solubility (WS) increased gradually with TME content, rising from approximately 40% for the control to about 55% for the 3% TME-SPI film, the water vapor permeability (WVP) concurrently decreased from about 55% to approximately 40% over the same concentration range.

This distinctive dual effect—concurrently increased hydrophilicity and improved moisture barrier—originates from the synergistic action of the TME within the SPI matrix. The hydrophilic components of the microemulsion, specifically Tween 80 and ethanol, increase the overall polarity of the film matrix, thereby enhancing its water affinity and WS. Concurrently, the uniformly dispersed nanodroplets are firmly embedded and act as impermeable physical fillers within the continuous SPI gel network, creating a highly tortuous pathway (the labyrinth effect) for water vapor molecules attempting to permeate through the hydrophilic matrix. This tortuosity notably reduces the WVP. This outcome is particularly noteworthy, as numerous studies indicate that the incorporation of hydrophilic modifiers frequently causes a rise in WVP, undermining the moisture-proof function. The capability of this TME-SPI system to improve the moisture barrier even within alkaline (pH 9) film-forming highlights its distinct advantage and practical potential for applications requiring controlled humidity exchange, like in the packaging of intermediate-moisture food products.

##### Mechanical Characteristics: A Smart Shift from Brittleness to Toughness

As illustrated in Figure 4d, the addition of TME markedly altered the mechanical properties of the films. Tensile strength (TS) decreased moderately from approximately 12 MPa (control) to approximately 8 MPa (3% TME-SPI) as the TME content increased. In comparison, the elongation at break (EAB) showed a pattern of initially rising and then falling. The 2% TME-SPI film exhibited the maximum EAB of 144%, approximately three times that of the control film (55%).

This shift from brittle to ductile mechanical behavior was attributed to the dual function of TME within the SPI gel matrix. First, Tween 80 functions as a small-molecule plasticizer, intercalating between protein chains to reduce intermolecular forces and enhance chain mobility. Second, the uniformly dispersed soft nanodroplets function as stress-dissipating centers, promoting craze formation and shear banding to dissipate fracture energy more efficiently. Compared to recently reported SPI-based active packaging films, which typically exhibit a trade-off between strength and flexibility, the current system attains a remarkably high elongation (greater than 140%) while maintaining a moderate tensile strength (greater than 8 MPa). This shows a more well-balanced mechanical profile that is suitable for flexible packaging uses. To evaluate the mechanical performance of the TME-SPI films within the context of the current literature, a comparison with recently reported SPI-based active packaging films is warranted. Huo et al. [19] developed SPI emulsion films incorporating green Sichuan pepper essential oil (GSPO) and reported that the tensile strength increased from 1.16 MPa (SPI film) to 1.48 MPa at the optimal GSPO loading of 1.0 g, while the elongation at break increased from 122% to 277%. The observed increase in both TS and EAB was attributed to the plasticizing effect of the essential oil and the formation of hydrogen bonds between GSPO and SPI, which enhanced the structural integrity of the film matrix. The mechanical profile of the SPI-GSPO films reported by Huo et al. [19] is characterized by high extensibility but relatively low tensile strength. In the present study, the 2% TME-SPI film achieved an EAB of 144% while retaining a TS above 8 MPa, representing a more balanced mechanical profile that combines moderate strength with substantial extensibility. The simultaneous achievement of moderate tensile strength and high elongation is attributed to the synergistic action of two distinct mechanisms: Tween 80 acting as an efficient plasticizer to enhance protein chain mobility, and the embedded nanodroplets functioning as stress-dissipating elements that promote craze formation and shear banding without compromising structural integrity. This unique combination of toughening mechanisms is intrinsic to the microemulsion-in-gel approach and clearly distinguishes the TME-SPI system from conventional emulsion-based composite films.

### 2.3. Antioxidant Activity and Sustained-Release Properties

**Figure 5:**
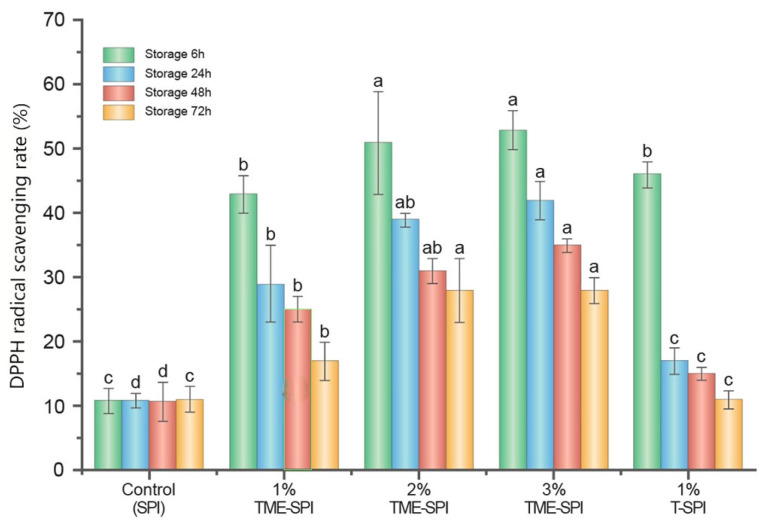
DPPH radical scavenging rate (%) of SPI films incorporated with TTO microemulsion (TME) and free TTO over 72 h. Different lowercase letters at the same time point indicate statistically significant differences (*p* < 0.05) between formulations.

The DPPH radical scavenging activity of the gel films over 72 h is presented in Figure 5, which reveals markedly different release dynamics among the formulations. All gel films that included either free TTO or TME showed notably improved antioxidant activity when compared to the control during the initial 6 h. Notably, the 1% T-SPI film (containing free TTO) exhibited the highest initial activity of approximately 45% at 6 h, outperforming the 1% TME-SPI film at this initial time point. However, the release profiles diverged considerably over time. The 1% T-SPI film showed a distinct burst release, and its activity quickly declined to under 10% after 72 h. In comparison, the TME-included formulations (1–3% TME-SPI) showed a more regulated and long-lasting release pattern [26]. Specifically, the 2% TME-SPI film retained 27.4% of its scavenging activity after 72 h, compared with its initial value of 51.8% at 6 h. Both the 2% TME-SPI and 3% TME-SPI films maintained good antioxidant capacity after 72 h, and there were no statistically notable differences between them at any time juncture.

The sustained antioxidant activity of the TME-SPI films can be attributed to two complementary mechanisms operating at different length scales: the molecular-level radical scavenging capability of the TTO constituents, and the nanoscale structural control exerted by the microemulsion-gel architecture on the release kinetics.

At the molecular level, the primary antioxidant constituents of TTO are monoterpenes and their oxygenated derivatives, with terpinen-4-ol, γ-terpinene, and α-terpineol representing the most abundant bioactive compounds. These molecules function as effective radical scavengers through a hydrogen atom transfer (HAT) mechanism, in which the phenolic hydroxyl groups of terpinen-4-ol and α-terpineol, or the allylic hydrogen atoms of γ-terpinene, react with the stable DPPH radical, reducing it to the non-radical DPPH-H form and thereby neutralizing its activity. The high initial scavenging rates observed in both TME-SPI and T-SPI films during the first 6 h confirm the intrinsic radical scavenging potency of the TTO constituents.

At the nanoscale, the microemulsion architecture embedded within the SPI gel matrix establishes a dual-barrier release control system that governs the long-term release kinetics. The Tween 80 interfacial film surrounding each oil droplet serves as the primary diffusion barrier. The densely packed surfactant monolayer imposes a partitioning step, requiring antioxidant molecules to first desorb from the oil core into the interfacial region before diffusing into the surrounding aqueous/hydrogel medium. This interfacial resistance substantially retards the initial release rate compared with the unimpeded volatilization and diffusion characteristic of free TTO. Subsequently, the continuous SPI gel network constitutes a secondary transport barrier. The physically crosslinked protein hydrogel forms a highly tortuous diffusion pathway (the labyrinth effect) through which antioxidant molecules must navigate. The effective diffusivity ($D_{eff}$) of the antioxidant through this composite barrier is considerably lower than its diffusivity in a homogeneous liquid medium, consistent with Fick’s second law incorporating a reduced effective diffusion coefficient:

$$\frac{\partial C}{\partial t}=D_{eff}\frac{\partial^{2}C}{\partial x^{2}}$$

where $D_{eff}$ is governed by both the interfacial partitioning coefficient at the oil/surfactant/water boundary and the tortuosity factor of the gel network.

In contrast, the 1% T-SPI film, which lacks this hierarchical encapsulation architecture, exhibited a characteristic burst release profile. The unprotected TTO was immediately available for volatilization from the film surface and rapid diffusion through pores or defects within the protein matrix, resulting in a precipitous decline in antioxidant capacity from approximately 45% at 6 h to below 10% within 72 h. The stark contrast between the burst release of free TTO and the sustained release from the TME-SPI system—in which the 2% TME-SPI film retained over 50% of its initial activity after 72 h—provides compelling evidence for the effectiveness of the microemulsion-based nanoencapsulation strategy. Furthermore, the observation that the 2% and 3% TME-SPI formulations exhibited statistically equivalent antioxidant activity at all time points (*p* > 0.05) indicates that a TME loading of 2% is sufficient to achieve the maximum practical antioxidant benefit, beyond which additional encapsulated TTO does not measurably enhance radical scavenging activity under the tested conditions. This finding carries practical implications for optimizing the cost-effectiveness of the composite film formulation.

The release kinetics of antioxidants were highly reliant on whether TTO was encapsulated in the microemulsion. At the beginning, the 1% T-SPI film showed greater activity than the TME-containing ones, which could be ascribed to its higher content of easily accessible, non-encapsulated TTO. However, this free TTO resulted in a typical burst release, rapidly depleting the active ingredient. In comparison, every TME-SPI film showed sustained release patterns. This results from the efficient nano-encapsulation of TTO in the TME. The oil core functions as a reservoir for the hydrophobic active substances. Meanwhile, the Tween 80/ethanol interfacial film acts as a diffusion obstacle, notably retarding the diffusion of antioxidants into the surrounding medium. This is a mechanism in line with Fickian diffusion combined with interfacial retardation [27]. Both the 2% and 3% TME-SPI films offered strong antioxidant protection even after 72 h, demonstrating the effectiveness of the microemulsion-based approach in prolonging antioxidant activity.

In comparison with other reported systems, the long-term release effect achieved in this case is remarkable. While gel films containing physically mixed essential oils frequently lose more than 80% of their activity within 24 h [28], the 2% TME-SPI formulation maintained over 50% of its starting activity after 72 h. This enduring activity holds substantial practical worth. The sustained release of antioxidants can effectively prolong the shelf life of foods prone to oxidation, such as lipid-rich and meat items. Moreover, considering the similar performance of the 2% and 3% TME-SPI films, the 2% formulation presents the greatest practical application value in view of economic cost.

### 2.4. Antibacterial Activity of Composite Gel Films

The antibacterial activity of the gel films against Staphylococcus aureus, a common Gram-positive foodborne pathogen, was evaluated by the colony counting method. The results, presented in Table 2, demonstrate a distinct concentration-dependent antibacterial effect for the TME-SPI films. The neat SPI film significantly reduced the colony count from 702 ± 42 CFU/plate (blank control) to 423 ± 38 CFU/plate (*p* < 0.05), exhibiting a baseline inhibition rate of approximately 40%. This inherent antibacterial activity likely originates from the alkaline film-forming environment and/or the presence of specific peptide fragments or other components within the SPI matrix that possess mild antibacterial properties.

The incorporation of 1% free tea tree oil (1% T-SPI) resulted in a colony count of 492 ± 39 CFU/plate, which was slightly but significantly higher than that of the neat SPI film (423 ± 38 CFU/plate, *p* < 0.05). This observation confirms that the simple addition of TTO without a pre-encapsulation strategy does not translate into enhanced antibacterial performance and can even compromise the intrinsic activity of the matrix, likely due to poor dispersion and the formation of heterogeneous aggregates. In stark contrast, the TME-SPI series exhibited a significant and dose-dependent enhancement in antibacterial activity. The colony counts were significantly reduced to 308 ± 31, 155 ± 19, and 125 ± 15 CFU/plate for the 1%, 2%, and 3% TME-SPI films, respectively (all *p* < 0.05 vs. blank control and vs. neat SPI). The 3% TME-SPI film achieved a remarkable 82% inhibition rate relative to the blank control, representing a more than threefold increase in antibacterial efficacy compared with the neat SPI film and substantially outperforming the free TTO-loaded film.

The mechanism underpinning this significantly enhanced antibacterial activity is attributed to the nano-encapsulation architecture of the TME. The microemulsion system ensures a homogeneous dispersion of TTO within the SPI hydrogel matrix as nanoscale droplets with a high surface-to-volume ratio. This facilitates maximum contact between the TTO and bacterial cells. The antibacterial action is presumed to proceed via the disruption of the bacterial cell membrane’s structural integrity by the bioactive terpenes (e.g., terpinen-4-ol), leading to the leakage of cytoplasmic contents and cell death. The sustained contact afforded by the gel film matrix, in conjunction with the high effective surface area of the nanodroplets, amplifies this destructive effect in a concentration-dependent manner. These results directly and convincingly demonstrate that the composite film itself, as a complete system, possesses a potent and tunable antibacterial activity, successfully addressing the critical validation requirement.

## 3. Conclusions

In this study, a multifunctional active packaging composite gel film was successfully fabricated by integrating an optimized tea tree oil microemulsion (TME) into a soy protein isolate (SPI) hydrogel matrix through a microemulsion-in-gel approach. The TME demonstrated excellent colloidal stability under various stress conditions, including dilution, thermal treatment, pH variation, centrifugation, and long-term storage, thereby serving as a robust carrier for subsequent film preparation. Upon incorporation of the TME into the SPI matrix, the resulting composite films exhibited a markedly enhanced light-barrier capability, as evidenced by increased opacity and reduced light transmittance. The hydration behavior displayed a favorable dual effect: water solubility increased moderately owing to the hydrophilic surfactant components, while water vapor permeability was notably reduced due to the tortuous-path effect imposed by the uniformly dispersed nanodroplets within the gel network. Mechanically, the films underwent a transition from brittle to ductile behavior, achieving substantially improved elongation at break while maintaining adequate tensile strength. Most significantly, the microemulsion encapsulation strategy imparted sustained antioxidant release to the composite films, enabling prolonged radical scavenging activity over 72 h, in stark contrast to the rapid burst release and sharp activity decline observed in films loaded with free tea tree oil. These findings collectively demonstrate that the microemulsion-in-gel approach effectively resolves the compatibility challenge between hydrophobic active compounds and hydrophilic protein matrices, enabling the simultaneous enhancement of light-barrier properties, moisture resistance, mechanical flexibility, and long-lasting antioxidant functionality. Furthermore, the composite films exhibited potent, concentration-dependent antibacterial activity against *S. aureus*, achieving an 82% inhibition rate at 3% TME loading, thereby providing direct experimental validation of their active packaging functionality. Overall, this strategy provides a promising framework for the design of next-generation high-performance active food packaging materials, and future work should prioritize evaluating the antibacterial efficacy of these composite films against common foodborne pathogens and assessing their practical performance in extending the shelf life of real food products under simulated storage conditions.

## 4. Materials and Methods

### 4.1. Materials

SPI, used as the gelling matrix for film formation, was obtained from Shandong Yuwang Ecological Food Industry Co., Ltd. (Dezhou City, China). Tea tree oil was procured from Jian Huaxin Natural Plant Co., Ltd. (Ji’an City, China), and 1,1-diphenyl-2-picrylhydrazyl was obtained from Hefei BASF Biotechnology Co., Ltd. (Hefei City, China). All other chemical reagents were acquired from Sinopharm Chemical Reagent Co., Ltd. (Shanghai City, China). All experiments were performed using double-distilled water prepared in our laboratory.

### 4.2. Preparation of TTO Microemulsion

Pseudo-ternary phase diagrams were constructed to identify the optimal microemulsion region [29]. Briefly, Tween 80 and ethanol were mixed at mass ratios of 1:1, 1:2, and 2:1 to prepare the surfactant/cosurfactant mixture (Smix). For every Smix ratio, TTO and Smix were mixed at mass ratios ranging from 9:1 to 1:9 in 1:1 increments. For every oil-to-Smix ratio, two ternary mixtures (5 g each) were prepared. Subsequently, while under magnetic stirring at a temperature of 25 ± 1 °C for 15 min, the aqueous phase was added dropwise into one of the two mixtures for each ratio until the system changed from clear to somewhat turbid. The quantity of the aqueous phase added at this termination point was noted. Data from all tested ratios were compiled to construct the phase diagrams and identify the optimal microemulsion composition for subsequent incorporation into the SPI gel matrix.

### 4.3. Preparation of SPI Gel Films

Based on the overall pseudo-ternary phase diagram examination, the SPI gel film-forming solution (the precursor to the gel network) was prepared in the following way [29]. Specifically, TTO, ethanol, and Tween 80 were placed in a beaker at a mass ratio of 2:9:9. The mixture was stirred continuously for 2 h using a magnetic stirrer to ensure complete homogenization. After agitation, the beaker was left undisturbed in a dark environment for 24 h to enable the formation of a stable TTO self-microemulsifying system, which was then utilized in the film preparation.

The SPI gel films were fabricated by a one-pot method followed by solution casting. The particular formulations of the film-forming solutions are presented in Table 3.

Using the 1% TME-SPI film as a representative example, the preparation procedure was as follows. SPI (5 g) was dissolved in 100 mL of distilled water under continuous stirring and heated to 80 °C to induce protein unfolding, a prerequisite for the formation of a homogeneous gel network. Subsequently, glycerol (1.5 g) was added and stirred for 10 min, after which the self-microemulsifying system (TME, 2 g) was introduced and stirring was continued for an additional 2 h to ensure uniform distribution of the nanodroplets throughout the viscous gel-forming liquid.

The pH of the gel-forming solution was then adjusted to 9 by the dropwise addition of NaOH solution (0.1 mol/L) while continuously stirring, and the stirring was continued for another 20 min to facilitate the formation of a homogeneous gel network structure prior to casting. The suspension was filtered by vacuum filtration and degassed using a rotary vacuum evaporator at 40 °C until no bubbles remained.

Afterward, a 20 mL aliquot of the gel-forming solution was cast onto an acrylic plate and spread uniformly using a casting blade. The cast solution was placed in a vacuum drying oven at 45 °C for 20 h, during which time the SPI sol–gel transition was completed and the gel network gradually dehydrated to yield a free-standing gel film. Following the dehydration process, the resulting gel film was carefully peeled from the plate. Finally, the stripped gel film was conditioned at 23 °C and 50% relative humidity for 24 h prior to testing to equilibrate the moisture content within the gel matrix. All other SPI gel films were fabricated following the same procedure.

### 4.4. Characterization of Microemulsions

#### 4.4.1. Particle Size and Polydispersity Index

The microemulsion was diluted 2-, 10-, 50-, and 100-fold with distilled water [30]. The mean particle size (Z-average) and polydispersity index (PDI) of the diluted specimens were measured at 25 °C via a laser particle size analyzer (Nano-ZS, Malvern Instruments, Worcestershire, UK). All measurements were carried out in triplicate to guarantee statistical dependability.

#### 4.4.2. Stability Studies

The physical stability of the microemulsion was evaluated under the following stress conditions: thermal stress (incubation at 25–60 °C for 20 min), long-term storage (25 °C in the dark for up to 30 days), pH stress (adjustment to pH 5–10 with 0.5 mol/L NaOH), and centrifugal stress (centrifugation at 1000–4000 rpm for 20 min).

For all conditions, the stability was evaluated by monitoring changes in the particle size and polydispersity index (PDI), as well as by visual inspection, for signs of phase separation, cloudiness, or precipitation.

### 4.5. Characterization of SPI Gel Films

#### 4.5.1. Appearance and Color

The appearance and color of the SPI gel films were assessed by means of a colorimeter (NR110, Shenzhen ThreeNH Technology Co., Ltd., Shenzhen City, China). The values of L (lightness), a* (red-green), and b* (yellow-blue) were noted. The total color difference (ΔE) relative to the control film (Control-SPI) was calculated as follows:

$$\Delta E=\sqrt{{\left(L^{*}-L\right)}^{2}+{\left(a^{*}-a\right)}^{2}+{\left(b^{*}-b\right)}^{2}}$$

where L, a, and b are the color parameters of the control SPI film (dimensionless units).

#### 4.5.2. Thickness

The thickness of the film specimens was measured by means of a digital micrometer (Mitutoyo, Kawasaki, Japan) having an accuracy of 0.001 mm. To guarantee precision, measurements were taken at five randomly chosen spots on each film, and the mean value was subsequently calculated for additional analyses.

#### 4.5.3. Water Solubility (WS)

The water solubility of SPI gel films was determined by the approach described in a recent report. The composite film was cut into 20 mm × 20 mm squares and put into a clean beaker. After being dried in an oven at 100 °C until reaching a constant weight, the initial mass of the film (M) was noted.

An appropriate quantity of water was added to the beaker. The beaker was kept at room temperature for 12 h, after which the water was removed. The beaker was dried once more in an oven at 100 °C until a constant weight was achieved, and the mass m of the film was measured. The solubility in water was calculated by using the subsequent formula:

WS = (M − m)/M × 100%.

where M represents the initial mass (g), and m stands for the mass after drying (g).

#### 4.5.4. Water Vapor Permeability (WVP)

The permeability of water vapor in the films was measured by the gravimetric approach as per ASTM E96 [31]. The film specimen was sealed using Vaseline at the opening of a glass test tube holding 9 g of anhydrous CaCl_2_ (0% RH) and subsequently put into a desiccator kept at 25 °C filled with a saturated KBr solution (84% RH). The increase in cup mass was noted every 24 h for a period of 7 days. The WVP was calculated by using the subsequent formula:

$$WVP=\frac{(m2-m1)\times d}{A\times t\times ∆P},$$

where m1 and m2 signify the mass prior to and subsequent to permeation (g), d stands for the membrane thickness (mm), A represents the effective area of the membrane (m^2^), t denotes the time (h), and ΔP is the vapor pressure difference (kPa) [25,26]. The resulting WVP is expressed in g·mm·m^−2^·h^−1^·kPa^−1^.

#### 4.5.5. Optical Properties

The light-related properties of the films were assessed by means of a UV-Vis spectrophotometer. Film specimens were sliced into rectangular shapes and positioned directly adjacent to the inner wall of a quartz cuvette. A blank cuvette was employed as a reference. The absorbance at 600 nm (A_600_) was noted. The opaqueness (T) and transmittance (Tr) were calculated as follows:

$$\begin{array}{c} T=\frac{A_{600}}{d}\\ Tr=10^{-A_{600}} \end{array}$$

In the formula, A_600_ represents the absorbance at 600 nm (dimensionless); d denotes the thickness of the film (mm); and Opacity (T) is expressed in mm^−1^. Transmittance (Tr) is dimensionless and may be expressed as a fraction or percentage [22,26].

#### 4.5.6. Mechanical Properties

The tensile strength (TS) and elongation at break (EAB) of the films were determined via a texture analyzer (TA. XT Plus, Stable Micro Systems, Godalming, UK) in line with the ASTM D882 standard procedure [32]. The film strips were sliced into rectangular samples (50 mm × 10 mm) and elongated at a velocity of 60 mm/min. TS and EAB were calculated by the subsequent formulas:

$$TS=\frac{F}{d\times W}\;EAB=\frac{L_{1}-L_{0}}{L_{0}}\times 100\%$$

where F represents the maximum tensile force (N), d stands for the thickness of the membrane (mm), W denotes the width of the membrane (mm), and TS is expressed in MPa. L_0_ is the starting length (mm), and L_1_ is the length at the point of fracture (mm). EAB is expressed as a percentage (%) [21,25,26].

#### 4.5.7. Antioxidant Activity

The antioxidant activity of the SPI gel films was assessed by the DPPH radical scavenging assay, adapted from previously established methods with modifications for film analysis. Gel film samples (2 g) were cut into small pieces and either dissolved or dispersed in 30 mL of distilled water under stirring for 30 min. Afterward, 30 mL of ethanol was introduced, and the blend was homogenized by vortexing for 2 min. The mixture was then centrifuged at 4000 rpm for 20 min to remove insoluble residues, and the supernatant was collected for analysis.

A 5 mL aliquot of the supernatant was mixed with 5 mL of a newly prepared 100 milligrams per liter DPPH ethanol solution. The blend was fully vortex-mixed and incubated in the dark at room temperature for 30 min. Following the incubation process, the absorbance was determined at 517 nm with a UV-Vis spectrophotometer (T6 New Century, Beijing Purkinje General Instrument Co., Ltd., Beijing, China).

A blank specimen, composed of the film extract combined with pure ethanol, and a control specimen, composed of the DPPH solution combined with ethanol, were prepared simultaneously to correct for background absorbance.

The scavenging rate of DPPH radicals was calculated based on the subsequent formula:

$$DPPH\;Scavenging\;Rate\left(100\%\right)=\frac{A_{0}-(A_{i}-A_{j})}{A_{0}}\times 100\%$$

where A_1_ is the absorbance of the control (DPPH solution, ethanol), A_2_ is the absorbance of the sample (film extract, DPPH solution), and A_3_ is the absorbance of the film extract without DPPH (background absorbance). All absorbance values are dimensionless. The DPPH scavenging rate is expressed as a percentage (%).

To assess the long-term release of antioxidants from the films, the test was carried out on films that were pre-treated for 6, 24, 48, and 72 h at 23 °C and 50% relative humidity prior to extraction and analysis [20,25,26].

#### 4.5.8. Antibacterial Activity

The antibacterial activity of the gel film-forming solutions was evaluated against *S. aureus* using the colony counting method. The bacterial strain was activated in Luria-Bertani (LB) broth at 37 °C for 24 h. The activated culture was diluted 1000-fold with phosphate-buffered saline (PBS, pH 7.4) to obtain a bacterial suspension. An aliquot (0.1 mL) of this suspension was spread uniformly onto the surface of an LB agar plate. Subsequently, 0.5 mL of each gel film-forming solution (neat SPI, 1% T-SPI, 1% TME-SPI, 2% TME-SPI, and 3% TME-SPI), prepared as described in Section 4.3, was gently and evenly introduced onto the inoculated plates. A plate receiving only the bacterial suspension served as the blank control. All plates were prepared in triplicate and incubated upside down at 37 °C for 24 h. After incubation, the colony-forming units (CFU) on each plate were counted, and the antibacterial activity was expressed as the percentage inhibition relative to the blank control.

### 4.6. Statistical Analysis

All experiments were performed in triplicate, and results are expressed as mean ± standard deviation (SD). Data were analyzed by one-way analysis of variance (ANOVA) using SPSS 18.0, followed by Duncan’s multiple range test. A significance level of *p* < 0.05 was applied throughout the study. In all figures and tables, different lower-case letters (e.g., a, b, c, d) denote statistically significant differences between groups (*p* < 0.05), whereas the same letter or overlapping letters indicate no significant difference (*p* > 0.05). Key pairwise comparisons are also explicitly reported in the text with their corresponding *p*-values. Graphs were generated using Origin 2022 and R 3.9.0 software.

## Figures and Tables

**Table 1:** Effect of TTO, ME and TTO Additives on Color Properties of SPI Films.

Sample Number	L	a	b	ΔE
control (SPI)	37.84 ± 0.26 ^c^	2.37 ± 0.04 ^d^	5.80 ± 0.13 ^d^	0
1% TME-SPI	42.11 ± 0.77 ^b^	2.51 ± 0.06 ^bc^	16.51 ± 0.08 ^b^	11.53 ^b^
2% TME-SPI	45.19 ± 2.53 ^b^	2.56 ± 0.22 ^b^	16.39 ± 0.39 ^b^	12.89 ^b^
3% TME-SPI	50.72 ± 8.76 ^a^	3.77 ± 1.21 ^a^	21.41 ± 3.22 ^a^	20.28 ^a^
1% T-SPI	38.24 ± 1.22 ^c^	2.44 ± 0.77 ^cd^	6.25 ± 0.03 ^c^	0.61 ^c^

TME, tea tree oil microemulsion; TTO, tea tree oil; T-SPI, SPI film with free TTO; L, lightness; a, redness-greenness; b, yellowness-blueness; ΔE, total color difference. Different superscript letters in the same column indicate statistically significant differences (*p* < 0.05).

**Table 2:** Antibacterial activity of SPI-based composite gel films against *Staphylococcus aureus*.

Sample	Colony Count (CFU/Plate)	Inhibition Rate (%)
Blank Control	702 ± 42 ^a^	0
Neat SPI	423 ± 38 ^b^	39.7
1% T-SPI	492 ± 39 ^c^	29.9
1% TME-SPI	308 ± 31 ^d^	56.1
2% TME-SPI	155 ± 19 ^e^	77.9
3% TME-SPI	125 ± 15 ^f^	82.2

Values are presented as mean ± standard deviation (*n* = 3). Different superscript letters in the same column indicate statistically significant differences (*p* < 0.05).

**Table 3:** Formulations of SPI-Based Gel Film-Forming Solutions.

Sample Number	SPI% (*w*/*v*)	TTO% (*w*/*v*)	Tween 80% (*w*/*v*)	Anhydrous Ethanol (*w*/*v*)	Glycerol% (*w*/*v*)
Control (SPI)	5	0	0	0	2.5
1% TME-SPI	5	1	4.5	4.5	2.5
2% TME-SPI	5	2	9	9	2.5
3% TME-SPI	5	3	13.5	13.5	2.5
1% T-SPI	5	2	0	0	2.5

SPI, soy protein isolate; TTO, tea tree oil; TME, tea tree oil microemulsion; T-SPI, SPI film with free TTO.

## Data Availability

All data and materials are available upon request from the corresponding author.
